# Seasonal Dynamics of Fungi Associated with Healthy and Diseased *Pinus sylvestris* Needles in Northern Europe

**DOI:** 10.3390/microorganisms9081757

**Published:** 2021-08-17

**Authors:** Ahto Agan, Halvor Solheim, Kalev Adamson, Ari Mikko Hietala, Leho Tedersoo, Rein Drenkhan

**Affiliations:** 1 Institute of Forestry and Rural Engineering, Estonian University of Life Sciences, 51006 Tartu, Estonia; kalev.adamson@emu.ee (K.A.); rein.drenkhan@emu.ee (R.D.); 2 Natural History Museum and Institute of Ecology and Earth Sciences, University of Tartu, 50411 Tartu, Estonia; leho.tedersoo@ut.ee; 3 Norwegian Institute of Bioeconomy, N-1431 Ås, Norway; halvor.solheim@nibio.no; 4 Norwegian Institute of Bioeconomy Research, 7734 Steinkjer, Norway; ari.hietala@nibio.no

**Keywords:** *Dothistroma septosporum*, *Lophodermium conigenum*, colonization threshold, foliage fungal richness, foliage canopy position, mycobiome, PacBio sequencing

## Abstract

The relationship between the ecological success of needle pathogens of forest trees and species richness of co-inhabiting endophytic fungi is poorly understood. One of the most dangerous foliar pathogens of pine is *Dothistroma septosporum*, which is a widely spread threat to northern European forests. We sampled two *Pinus sylvestris* sites in Estonia and two in Norway in order to analyse the relations between the abundance of *D. septosporum* and overall fungal richness, specific fungal species composition, time of season, needle age and position in the canopy. In both countries, the overall species richness of fungi was highest in autumn, showing a trend of increase with needle age. The overall species richness in the second-year needles in Estonia and third-year needles in Norway was similar, suggesting that a critical colonization threshold for needle shed in *P. sylvestris* is breached earlier in Estonia than in Norway. The fungal species richness in *P. sylvestris* needles was largely affected by *Lophodermium conigenum*. Especially in older needles, the relative abundance of *L. conigenum* was significantly higher in spring compared to summer or autumn. The timing of recruitment and colonization mechanisms of different foliage endophytes are shortly discussed.

## 1. Introduction

Fungal diversity in the foliage of conifers is affected by many biotic and abiotic factors, e.g., tree health, vegetation (the presence of other tree species, type of ground vegetation), latitude, climate and microclimate, needle age, position of needles in tree crown, etc. [1,2,3]. The fungal community is comprised of endophytes and epiphytes [4], and it can be difficult to differentiate one group from another, because some epiphytes can also colonize leaf/needle tissues internally, and endophytes in turn can have an epiphytic phase in their life cycle [5]. It has also become evident that traditional classification of oomycete and fungal phytopathogens into biotrophs, hemibiotrophs and necrotrophs is too general, and a more precise distinction is needed [6]. Furthermore, previous studies suggest that endophytes can be herbivore antagonists and enhance plant growth in both grasses and woody plants [7,8,9,10,11]. However, these largely experimental findings are difficult to verify in the frame of the disease triangle under the natural growing conditions characterized by multiple abiotic and biotic interactions [12].

During the last decade, it has become evident that it can be difficult to differentiate endophytes from necrotrophs due to certain species having the ability to switch between feeding modes depending on the growth conditions and health status of host species [13]. As Sieber [12] postulated, endophytes accelerate needle senescence once the density of colonization exceeds a certain threshold. Adverse conditions such as lack of light or low temperatures can facilitate colonization rate to a degree that leads to premature needle cast. There are also conifer-specific needle pathogens that inhabit only young first-year needles. For example, spruce-infecting *Chrysomyxa* spp. [14] and the larch needle cast fungus *Meria laricis* are able to infect needles of their host trees only during the first four weeks after needle emergence. Most needle endophytes can infect needles of all age classes, with the susceptibility of the needles and the frequency of colonization being considered to increase with needle age. Johnson & Whitney [15] showed that colonization of black spruce (*Picea mariana* (Mill.) Britton, Sterns & Poggenburg) needles by endophytic fungi increased from 4% in the current-year-needles to 90% in 3-year-old needles. In addition, there are examples of first-year needles of Sitka spruce (*Picea sitchensis* (Bong.) Carr.) and *Pinus strobus* L. having an extremely low amount of endophytes in the genera *Lophodermium* and *Hormonema* in comparison to older needles [16,17]. 

Previous studies using either fungal culturing or 454 sequencing of the ITS2 region have shown that sampling time and geographical location can be the most common drivers of fungal diversity and richness of *Pinus sylvestris* L. needles [1,2]. Overall fungal species diversity of *P. sylvestris* needles showed an increase along the north to south gradient in Fennoscandia [1,2] and from spring to autumn [1]. Taudiere et al. [3] concluded that richness of endophytic fungal communities in Corsican black pine (*Pinus nigra* J.F Arnold) was similar across sites and tree cohorts or needle location within the canopy (shade needles compared to the light needles) but differed significantly among forest patches and trees of different age. 

There are several needle pathogens threatening *P. sylvestris* in northern Europe, e.g., *Dothistroma septosporum* (Dorogin) M. Morelet and *Lophodermium seditiosum* Chevall. Dothistroma needle blight (DNB) is a dangerous foliar disease of pine caused by two species, *Dothistorma septosporum* and *Dothistroma pini* Hulbary [18,19]. While *D. septosporum* is more widely distributed across Europe, *D. pini* is present only in the southern and central parts of the continent [19]. The DNB agents are distributed around the world and infect ca. 110 species of Pinaceae, mostly *Pinus* spp. [19]. 

*Dothistroma septosporum* was first described in 1911 in Russia [20,21]. Since then, it has caused several disease outbreaks in the northern hemisphere, especially frequently from the beginning of 1990s [19,22]. In Estonia, the pathogen was first found on the non-native *Pinus nigra* in 2006 and a year after on the native *P. sylvestris.* By 2008, *D. septosporum* was recorded across the country on *P. sylvestris* [23]. In Norway, *D. septosporum* was first discovered in 2009 in the Troms county [24], this representing the northernmost record of *Dothistroma* species in the world. In Norway, the main damage caused by *D. septosporum* has been observed on trees growing along rivers in valley bottoms, predominantly on second and third-year needles [19]. In Estonia, like in the entire northern Europe, older needles are more prone to *D. septosporum* infection than current -year needles, whereas no correlation between disease severity and tree vicinity to waterbodies is recorded [23]. It is known that losses of productive foliage (the first and second-year needles) are accompanied by growth rate decline of *P. sylvestris* [25,26].

*Lophodermium* Chevall. is a well-studied fungal genus in the family Rhytismataceae (Rhytismatales, Ascomycota). Species within this genus are ecologically diverse even though they exploit a common habitat, i.e., pine needles [27]. *Lophodermium* species have been historically the most often isolated species from needles of *Pinus*, *Abies* and *Picea* [28]. More than 20 species of *Lophodermium* are known to inhabit coniferous trees and shrubs and most of them are primary saprotrophs that are responsible for early decomposition of shed needles [29]. Only one of them, *L. seditiosum*, is considered a major pathogen [29]. *Lophodermium seditiosum* is known to infect pine needles in summer and early autumn, and the infected needles are shed from trees in the following spring [30,31]. This species, first recorded in Estonia in 1856 [32], is common in areas where *P. sylvestris* is propagated [31]. Lophodermium needle cast is a serious foliage disease that affects both the height and radial increment of *P. sylvestris*, particularly in forest nurseries and young plantations [31,33]. It is a matter of debate whether the recent outbreaks of different needle pathogens (especially *L. seditiosum* and *D. septosporum*) have been influenced by changes in fungal species composition, forest management activities, distribution of susceptible host species, global climate change or by a combination of all these factors [19,34].

We used PacBio third-generation sequencing to analyze fungal species richness and composition in the needles of *P. sylvestris* in northern Europe to test the following hypotheses: (1) Fungal species richness depends on the site temperature sum, sampling season, needle canopy position, tree health and needle age; (2) *Dothistroma septosporum* and *Lophodermium* species are more abundant in needles from lower parts of the canopies compared to upper parts; (3) Owing to species-specific variation in sporulation regimes, the relative abundance of the dominant fungal species peaks at different times of the season.

## 2. Materials and Methods

### 2.1. Study Sites and Sampling

The Estonian sites represented the *Vaccinium* site type [35], situated in Haabsaare (N57.7591, E26.5038; 95 meters above sea level, m.a.s.l.) and Konguta (N58.2281, E26.1558; 35 m.a.s.l.), with effective temperature sums (base temperature +5 °C) of 1763 °C and 1711 °C, respectively, in 2014. The Haabsaare site represents a *P. sylvestris*-dominated stand, with a pine age of 5–20 years and height of 1–8 m. *Pinus sylvestris* is present as both canopy forming and understory tree — the sampled DNB- symptomatic trees and healthy trees were growing in the understorey. The Konguta site is a pure *P. sylvestris* stand with 5–10- year-old (1–3 m) canopy-forming saplings. The Norwegian sites were located in Gransherrad (N59.6916, E9.04215, 188 m.a.s.l.) and Engerdal N61.7460, E11.9754, 544 m.a.s.l.), with effective temperature sums of 1590 °C and 895 °C, respectively, in 2014. The Gransherrad site was a pure stand of 3–6 m tall *P. sylvestris*, very dry with patches of understory vegetation dominated by *Calluna*. The Engerdal site was dominated by 3- to 12-m-tall *P. sylvestris*. The site was very heterogeneous, with boggy areas dominated by *Sphagnum* and dry areas characterized by *Vaccinium* in the ground vegetation.

Sampling was performed in summer (July) and autumn (October) of 2014 and in spring (April) of 2015 at the four above-mentioned sites in Estonia and Norway. In each site, two *P. sylvestris* trees with DNB symptoms and two trees with no foliage symptoms were selected and marked, this totaling eight healthy and eight diseased trees. The presence of typical DNB symptoms (red bands on needles) throughout the canopy was estimated visually during each sampling time. If no red bands were detected, the tree was considered healthy. From each sampled tree, the first- (needle-age-class 1) and second-year needles (needle-age-class 2) were sampled at each time point. In the case that third-year needles (needles of age-class 3) were present, these were also sampled. In the Estonian sampling sites, no third-year needles were retained in 2014. Although needles were sampled essentially from three different needle-age classes, the age of these needles within these classes differed depending on sampling time. In the spring 2015 (April), first-year needles were 11 months old, whereas in the summer (July) and autumn (September) 2014 first-year needles were 2 and 5 months old, respectively. Second-year needles were 23 months old in the spring 2015, and 14 and 17 months old in the summer and autumn 2014, respectively. Third-year needles were 26 and 29 months old in the summer and autumn 2014, respectively, and 35 months old in the spring 2015. From the 16 trees selected for sampling, the needles were collected separately from one randomly chosen alive branch from the third branch whorl, counting from the top of the tree canopy, and from one randomly chosen alive branch from the third branch whorl, counting from the base of the living canopy. This was done to consider potential qualitative or quantitative differences in fungal communities within tree crown. From each tree, three random needle pairs per needle-age class were sampled, cut into 0.5–1 cm pieces, placed into 2 mL Eppendorf tubes and stored in −20 °C for future analyses. The sampling scheme is illustrated in Figure S1.

### 2.2. Molecular Analysis

The needle tissue disruption was performed in a Retsch MM400 homogenizer (Retsch GmbH, Haan, Germany) using metal beads (2.5 mm), and DNA extraction was carried out using GeneJET Genomic DNA purification kit (Thermo Fisher Scientific, Vilnius, Lithuania), following the manufacturer’s instructions. For identification of fungi, the rDNA gene region ITS1-5.8S-ITS2 was amplified using the ITS1catta [36] and ITS4ngs [37] primers. The ITS1catta primer has been designed to exclude plant DNA and to improve the detection of fungal endophytes in woody plants. The 3′ end of the ITS4ngs primer was supplemented with a 10–12 base multiplex identifier (MID) tag. Each tag had at least four specific bases to allow sample identification. 

Conventional PCR was carried out according to Agan et al. [38]. The amplicons were pooled into two sequencing libraries (separately for each country) on an equimolar basis. The purification of PCR products was performed using GeneJet DNA purification kit (Thermo Fischer Scientific, Vilnius, Lithuania) following the manufacturer’s instructions. The protocols established for the RSII instrument of the PacBio third-generation sequencing platform (Pacific Biosciences, Inc. Menlo Park, CA, USA) were followed throughout the library preparation process. The diffusion method was used in loading the libraries to SMRT cells. Sequencing was performed using P6-C4 chemistry for 10 h following Tedersoo and Anslan [36] in University of Oslo in Norway.

### 2.3. Bioinformatics 

Bioinformatics was undertaken using the programs mothur [39], UCHIME [40], ITSx [41] and CD-Hit [42] as implemented in Pipecraft v1.0 [43]. Sequences were clustered into Operational Taxonomic Units (OTUs) based on 99% sequence similarity, because previous studies [44] showed that for separating Pinaceae-specific needle pathogens, such as *D. septosporum* and *D. pini*, a 99% sequence similarity cut-off is needed. The OTUs were taxonomically identified based on representative sequences using Pipecraft against UNITE v.7 database [45]. OTUs were considered as members of fungi if their representative sequences matched best fungal taxa at an e-value < e−50. Representative sequences that had >99% sequence similarity to reference sequences were assigned to species hypotheses (SH) based on UNITE v.7. Higher level classification of Fungi refers to Tedersoo et al. [46]. All OTUs that were originally identified either as *D. pini* or *Lophodermium* sp. were also manually checked against UNITE v.7 to reduce the risk of false identification. 

### 2.4. Statistical Analyses 

Richness of OTUs and rarefaction analyses were performed using PAST v3.25 [47]. Statistical calculations were performed in R studio version 1.1.456 using the package lme4 [48]. The square root of the total number of sequences per sample was added as a covariate, and tree identity was added as a blocking factor. The relationships of site, tree health, needle age, needle location in the canopy and date of sampling with the relative abundance of *D. septosporum* and *Lophodermium* spp. were tested using linear mixed models. Species richness and sequence read percentages of species were log-transformed prior to analyses.

To consider differences in fungal communities in relation to the experimental factors (site, tree health, needle age, needle location in the canopy and date of sampling) and their interactions, we used PRIMER v6 and PERMANOVA+ [49,50]. The species abundance matrix was square-root transformed to reduce the effect of dominant species. Bray-Curtis dissimilarity was used as a distance measure [51]. CAP (canonical analysis of principal coordinates), implemented in PRIMER v6 [49], was used in visualization of fungal community structure.

## 3. Results

### 3.1. Fungal Taxonomic Richness and Dominant Species 

The unfiltered dataset consisted of 58210 sequences, of which 2124 sequences were identified as Tracheophyta and removed from the dataset. After removing the low-quality sequences and singletons from the data set, 10176 full ITS sequences, retrieved from 190 samples and representing 261 OTUs, remained. From all the obtained ITS sequences, 55.7% represented Ascomycota, 42.5% Basidiomycota, 1.7% unidentified fungi and 0.2% Chytridiomycota. Most abundant OTUs in this dataset were assigned as *Lophodermium conigenum* (19.5% of all sequences), *Phacidiaceae* sp. (9.1%, extremely abundant in Norway, seldom found in Estonia), *Capnodiales* sp. (6.6%) and *Dothistroma septosporum* (6.4%). *Dothistroma septosporum* had two different OTUs in this dataset, the less abundant variant accounted for 0.1 % of all sequences in the dataset. There were considerable differences among the 20 most common fungal species between Estonia and Norway (Table S1).

Needles of *P. sylvestris* of different age harboured on average 9.3 (±0.64) (mean ± SE) fungal OTUs. On average, the Estonian sites Haabsaare and Konguta had 15.9 (±1.63) and 7.6 (±1.19) OTUs per sample, respectively, while the Norwegian sites Engerdal and Gransherrad had 5.8 (±0.62) and 8.9 (±1.28) OTUs per sample, respectively.

### 3.2. Seasonal Differences in Fungal Species Richness and Composition

There were marked differences in the relative abundance of phyla, orders, dominant species, and overall richness across sampling times (Figures S2 and S3). The relative abundance of all ascomycetes on pine needles dropped from 68.1% in spring to 50.4% in summer and to 50.1% in autumn. Of prevalent species, the relative abundance of *D. septosporum* increased from 4.3% in spring to 11.0% by summer and declined in autumn (3.7%). The relative abundance of *L. conigenum* was highest in spring (37.1%) and declined in summer (11.4%) and autumn (7.3%). The needle pathogen *Lophodermium seditiosum* was detected only in the Estonian samples at low abundance: 0.3% of all sequences.

**Figure 1:**
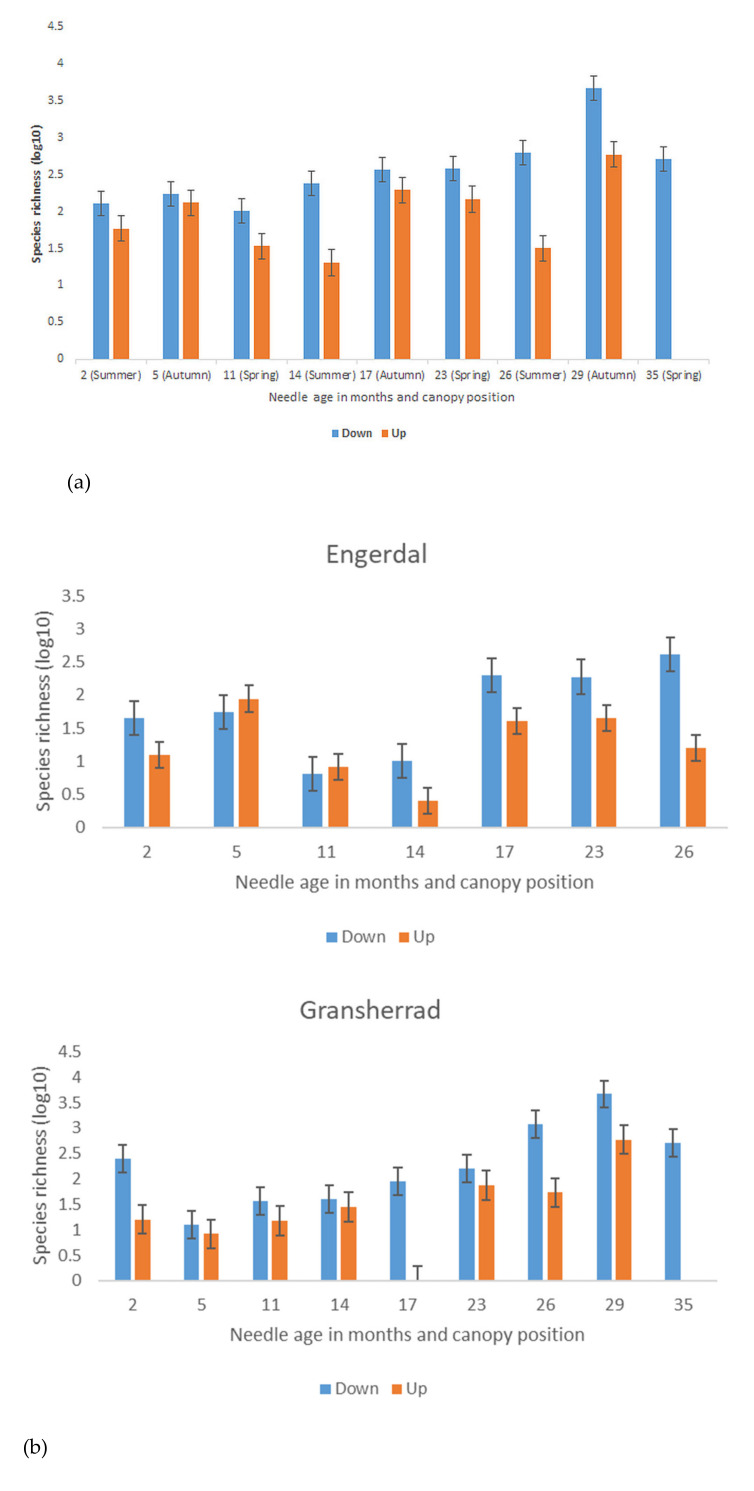

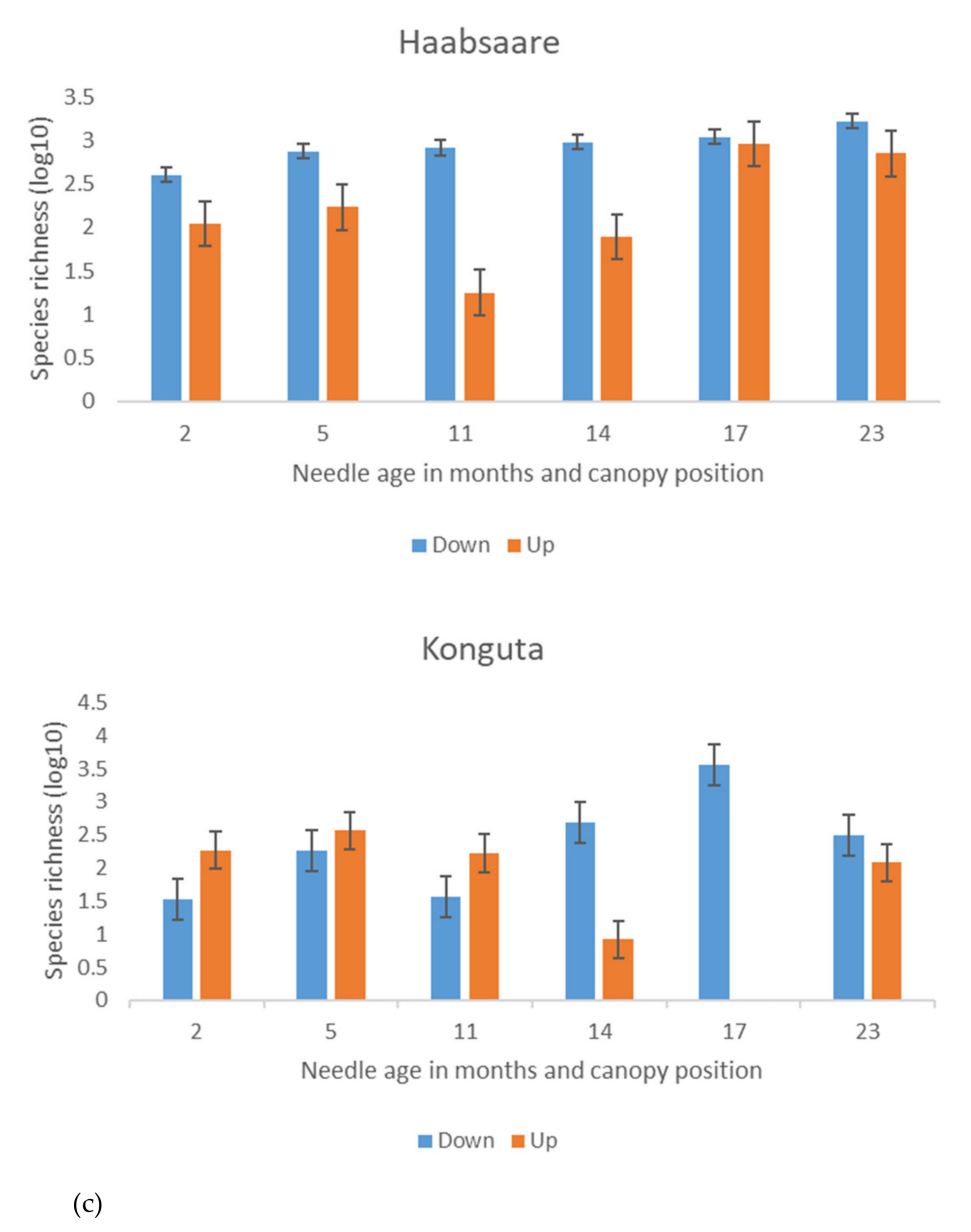
Overall fungal richness in *P. sylvestris* needles (n = 190) across needle-age cohorts in calendar months in all four sites combined (**a**); in Norway (**b**) and Estonia (**c**). The missing data for 17-month-old needles are due to technical errors in sequencing. Whiskers show standard error.

The constructed full model across two countries and four sampling areas showed that significant predictors of general species richness in *P. sylvestris* needles were country and sampling site (F_1.183_ = 37.7; R^2^_adj_ = 0.727; *p* < 0.05), species richness being significantly higher in Estonia than in Norway (Figure 1). Species richness was higher in autumn than spring (F_2.183_ = 17.1; R^2^_adj_ = 0.150; *p* < 0.05; Figure 1). Additionally, effective temperature sum had a significant positive relation with overall species richness (F_1.183_ = 11.0; R^2^_adj_ = 0.607; *p* < 0.01). A high sequence read percentage of *L. conigenum* (F_1.183_ = 16.7; R^2^_adj_ = 0.102 *p* < 0.001) was accompanied with lower general species richness. Needle age, presence of *D. septosporum* symptoms on tree and needle location in canopy did not have any significant (*p* > 0.05) relationship with overall fungal species richness in *P. sylvestris* needles. The two-year-old needles in Norway had significantly lower species richness when compared to needles of the same age in Estonia (F_1.86_ = 15.0; *p* < 0.001). While the needles were retained longer in the sampled trees in Norway compared to Estonia, the overall species richness of two-year-old needles in Estonia and three-year-old needles in Norway were similar (*p* > 0.05). 

As there were significant differences in fungal species richness between countries, we constructed separate models to determine the influencers of species richness within each country. We found that the significant drivers of species richness were sampling season (F_2.114_ = 2.45; R^2^_adj_ = 0.305; *p* < 0.05; Figure 1) and needle age class (F_2.114_ = 24.4; R^2^_adj_ = 0.201; *p* < 0.001; Figure 1) in Norway, but only sampling date had a significant effect on overall species richness in Estonia (F_2.83_ = 8.42; R^2^_adj_ = 0.657; *p* < 0.05; Figure 1).

### 3.3. Factors Affecting Relative Abundance of Pathogens D. septosporum and L. conigenum

**Figure 2:**
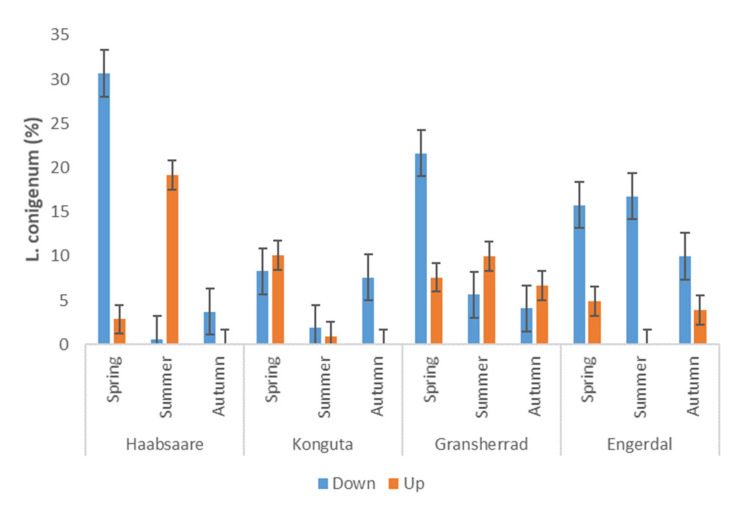
Sequence read percentage of *L. conigenum* on pine needles from two canopy positions across the three sampling times at the four sites (n = 190). Whiskers show standard error.

**Figure 3:**
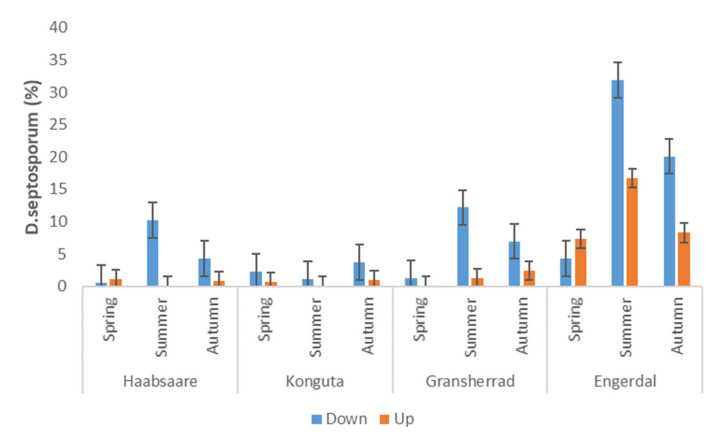
The relative abundance of *D. septosporum* on pine needles from two canopy positions across the three sampling times at the four sites (n = 190). Whiskers show standard error.

Sampling date was the main factor affecting relative abundance of *L. conigenum* in a full model considering both the Estonian and Norwegian samples (F_1.183_ = 7.53; R^2^_adj_ = 0.474; *p* < 0.001; Figure 2): the relative abundance of this species was significantly (*p* < 0.01) higher in spring when compared to summer and autumn. Conversely, the abundance of *D. septosporum* was low in spring at all four sites. In three sites (Engerdal, Gransherrad, and Haabsaare), *D. septosporum* showed its highest abundance in summer and a decline in autumn (Figure 3). In contrast, the Konguta site showed the highest percentage of *D. septosporum* in autumn compared to other sites. In a full model, the interaction of sampling site and sampling date had a significant influence on the abundance of *D. septosporum* (F_1.183_ = 1.53; R^2^_adj_ = 0.418; *p* < 0.05; Figure 2).

**Figure 4:**
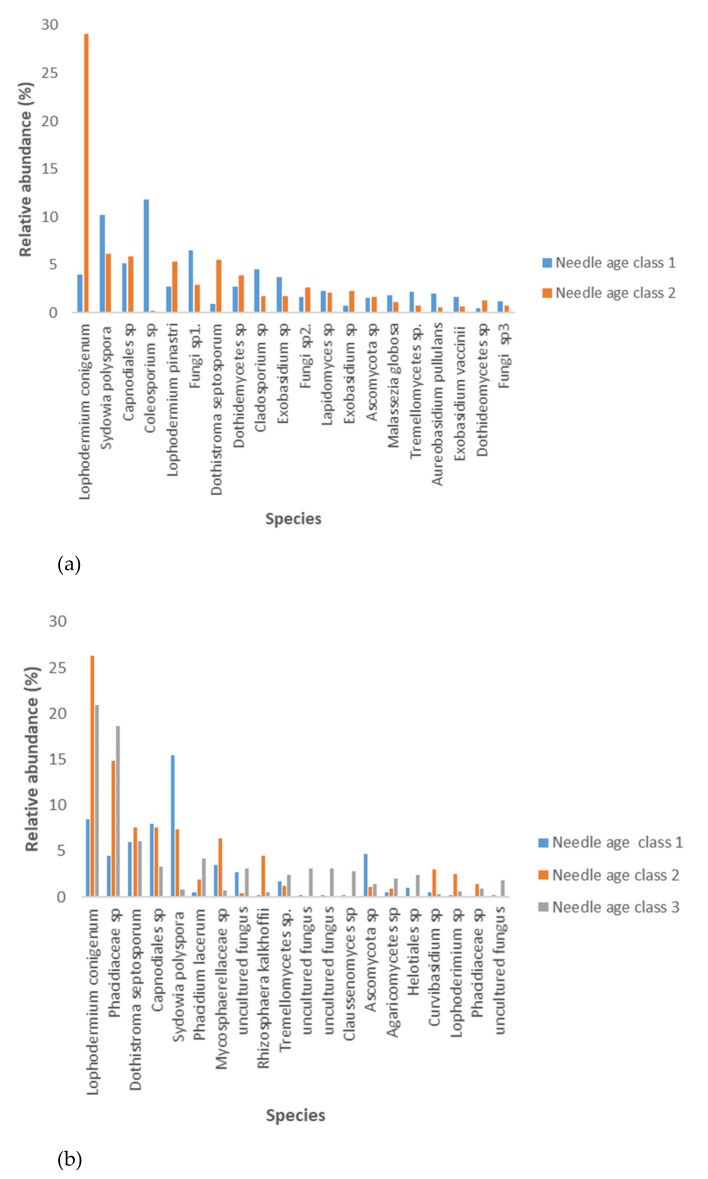
Twenty most abundant fungal OTUs in Estonia (**a**) and Norway (**b**) in needles with different ages (n = 190).

There was a positive association between needle age and the relative abundance of *L. conigenum* (F_2.183_ = 32.9; R^2^_adj_ = 0.411; *p* < 0.05) and that of *D. septosporum* (F_2.183_ = 14.9; R^2^_adj_ = 0.390; *p* < 0.05). Second-year and third-year needles harboured significantly (F_2.183_ = 5.30; R^2^_adj_ = 0.411; *p* < 0.05; Figure 4) more *D. septosporum* compared to first-year needles. The needle age, when expressed in months, associated positively with the percentage of *D. septosporum* (F_1.183_ = 5.43; R^2^_adj_ = 0.288; *p* < 0.01) but not with that of *L. conigenum*. At all sampling sites, the older needles also harboured a considerably higher percentage of *L. conigenum* in comparison to current-year needles (F_2.183_ = 7.85; R^2^_adj_ = 0.218; *p* < 0.01; Figure 4). On the current-year needles, the most prevalent fungal species were *Coleosporium* sp., *Sydowia polyspora* and *Phaeococcomyces* sp. (11.7%, 10.1% and 9.4%, respectively) in Estonia and *S. polyspora*, *Capnodiales* sp. and *D. septosporum* (15.5%, 7.5% and 6.0%, respectively) in Norway (Figure 4). 

The interaction of country and needle position within the canopy was associated (F_1.183_ = 1.86; R^2^_adj_ = 0.291; *p* < 0.01) with the relative abundance of *D. septosporum*: needles in the upper part of canopy had significantly lower sequence read percentages of *D. septosporum* than needles in the lower part in Norway. A similar trend was observed in Estonia, although it was not significant (*p* = 0.08). Relative abundance of *L. conigenum* was significantly lower in the upper-canopy needles than lower-canopy needles in both countries (F_1.183_ = 9.28; R^2^_adj_ = 0.218; *p* < 0.05).

### 3.4. Fungal Species Composition 

According to permutational ANOVA, the biggest influencer of species composition was sampling site (20.1%, *p* = 0.002), followed by needle location in the canopy and needle age (9.4% and 8.5%, respectively, *p* = 0.002). Sampling date explained 8.3% of the species composition (*p* = 0.01). Fungal species composition was also significantly (*p* = 0.004) different between healthy and diseased (DNB symptoms) trees. The interaction location x sampling date also had a significant effect on fungal species composition (*p* = 0.02). Other interactions with significant effect were sampling location x tree health (*p* = 0.02), sampling location x age of needles (*p* = 0.01) and needle location in canopy x tree health (*p* = 0.01), but these explained < 1% of the variation.

**Figure 5:**
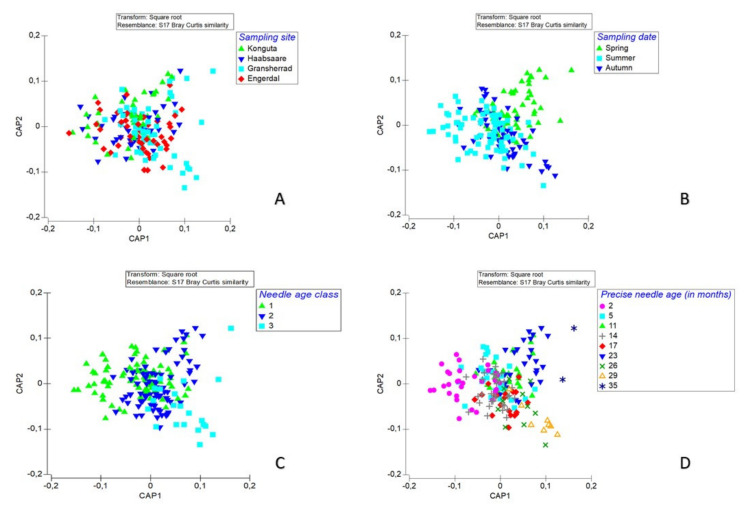
Canonical analysis of principal coordinates (CAP) of differences in fungal community structure between the four investigated sampling sites in northern Europe (two in Estonia, two in Norway); (**A**) three sampling dates (**B**), three needle-age classes (**C**) and precise needle ages in months (**D**).

CAP analysis (Figure 5) showed significant (*p* < 0.05) differences in species composition between the sampling sites, sampling dates and needle age classes.

Analysis of co-occurrence of species showed that out of 1127 interactions, 360 were positive, 4 were negative and 763 were random. *Lophodermium conigenum* had no negative interactions, but 31 positive interactions. *Dothistroma septosporum*, however, had two statistically significant negative associations, with *Chaetothyriales* sp. (*p* = 0.008) and *Malassezia restricta* (*p* = 0.03), and 29 positive interactions (Table S2). 

In order to consider the relations between relative abundance of *L. conigenum* and *D. septosporum* and fungal species richness, we constructed three models (one for each sampling time, data from Estonia and Norway combined). The results showed that *L. conigenum* had a significant negative association with fungal richness of pine needles in summer (F_1.183_ = 9.76; R^2^_adj_ = 0.328; *p* < 0.01) and autumn (F_1.183_ = 28.8; R^2^_adj_ = 0.525; *p* < 0.01) but not in spring even though the percentage of *L. conigenum* peaked at this time. The negative association of *L. conigenum* with the overall fungal species richness was significant in second-year needles of *P. sylvestris* in Estonia (F_2.89_ = 23.9; R^2^_adj_ = 0.861; *p* < 0.01) and in second- and third-year needles in Norway (F_2.69_ = 11.5; R^2^_adj_ = 0.668; *p* < 0.01). *Dothistroma septosporum* had no significant association with species richness for any of the sampling dates, sites or needle-age classes (*p* > 0.05).

## 4. Discussion

### 4.1. Fungal Taxonomic Richness

PacBio sequencing of 190 needle samples from four sampling sites revealed 261 OTUs, of which *L. conigenum*, *Phacidiaceae* sp., *Capnodiales* sp. and *D. septosporum* prevailed. The genus *Lophodermium* is especially diverse on the needles of pine and these species mostly function as endophytes and saprobes [1,2,3,52], except the pathogen *L. seditiosum*. 

*Lophodermium conigenum* was also one of the three most abundant species on 1-year-old needles of Corsican black pine in Corsica in a study based on metabarcoding of the ITS2 rDNA region, being present in all three of the investigated sites, although with variable abundance [3]. In another study utilizing metabarcoding of ITS2, needles of *Pinus heldreichii* H. Christ harboured *L. conigenum* as the second most prevalent species in six different sites in Montenegro mountains [52]. These authors concluded that *L. conigenum* was primarily associated with trees in good health, being one of the most dominant species in sites with moderate growth conditions and healthy trees, but with low abundance in areas where beetle attacks and necrotic lesions (agent unknown) on needles occurred. It is noteworthy that Lazarevic and Menkis [52] pursued the entire spectrum of needle-associated fungi and thus pooled together 2–4-year-old needles, both healthy looking and symptomatic ones, as a single sample per each experimental tree, this complicating direct comparisons with other studies. Based on a fungal isolation approach, *L. conigenum* was the most frequently isolated fungal OTU from symptomless needles of *P. sylvestris* in Finland [1]. Utilizing a metabarcoding approach, Millberg et al. [2] found that *L. conigenum* was more abundant on symptomatic needles of *P. sylvestris* in northern Sweden. However, the roles of *L. conigenum* in pine phylloplane remain to be clarified—but it seems to be an endophyte, detected in high frequency in recent mycobiome studies of different *Pinus* species in Europe, regardless of geographical location. *L. conigenum* has been proposed to act as an antagonist against *L. seditiosum* or *L. pinastri* s. str. [53]. 

*D. septosporum* has been seldom found [52] or not found at all in previous isolation- or sequencing-based studies on fungal diversity in pine foliage [1,2,3]. In the Nordic countries, the sampling in prior *P. sylvestris* foliage studies was performed during 2006–2008, at a time when *D. septosporum* was probably not widely spread in the region. In Finland and Sweden, the pathogen was first recorded in 2007 [54,55]. In our study, *D. septosporum* was relatively abundant across the entire dataset, representing 6.4% of all sequences, and found on both visually healthy and symptomatic trees. Species co-occurrence analysis showed that abundance of *D. septosporum* was positively associated with that of *L. conigenum*. Further studies, including dual culturing, are needed to assess the nature of this relationship.

### 4.2. Season-, Needle Age-, and Canopy Position-Related Differences in Fungal Species Richness

Fungal species richness was significantly higher in autumn than in spring or summer in both countries (Figure 1). Effective temperature sum had a significant (*p* < 0.01) positive association with overall fungal species richness, but it should be noted that inclusion of more sites would be needed to test whether this represents a true gradient or stochastic variation only. In Norway, where also 3-year-old needles could be sampled, needle age showed a positive relation with species richness (*p* < 0.001). The fungal species richness was significantly (*p* < 0.001) higher in second-year needles in Estonia compared to needles of the same age in Norway. In the frame of critical colonization threshold [12], this would suggest that the carrying capacity of a needle is reached earlier at the Estonian sites due to higher colonization density coupled with warmer temperatures when compared to the more northern Norwegian sites that are located also at a higher altitude with lower effective temperature sum. This warmer climate could also mean a higher colonization density and longer activity period of fungi in needles of *P. sylvestris* in Estonia, which in turn can trigger earlier needle senescence compared to Norway (Figure S4). Quantitative PCR or spike-in controls with a general fungal assay [56] would be needed to consider this hypothesis since metabarcoding can only estimate the relative abundance of different species.

An autumn increase in fungal species richness in the foliage of different deciduous tree species has been reported by several previous studies [38,57,58], where it was mostly explained by late-season foliage senescence [59]. In *P. sylvestris*, species richness increased during the last year before needle shed at Konguta (2-year-old needles) and Gransherrad (3-year-old-needles) sites especially in autumn (see Figure 1 and Figure S5). For Engerdal and Haabsaare, the data are inconclusive, possibly due to small sample size and stochastic variation. Similarly, Zamora et al. [60] found that the fungal species richness of needles of four different *Pinus* species were highest in autumn compared to spring in Spain. Our study showed no significant differences in overall fungal species richness between diseased and healthy *P. sylvestris* trees. This could result from some of the healthy trees being below a critical colonization threshold, above which DNB symptoms are triggered. As *D. septosporum* has a hemi-biotrophic lifestyle, the fungus spends the first part of its life cycle as an epiphyte on the surface of needles without causing any visible damage to needles [61]. After the epiphytic phase, *D. septosporum* colonizes first the epistomatal chamber, followed by mesophyll colonization and gradual mesophyll cell disintegration, and finally collapse of endodermal cells and appearance of fruiting bodies [61]. It is reasonable to presume that the characteristic red bands on needles, resulting from secretion of the toxin dothistromin by *D. septosporum* [22], appear once a specific colonization level is breached by the pathogen. Latent infection of *P. sylvestris* needles by *D. septosporum* was recorded in Sweden where the fungus was detected on several two-year-old seedlings without any symptom development [55]. The authors noted that while at some time points infection of *D. septosporum* was detected in more than 50% of the seedlings, red bands and conidiomata were evident only on a small proportion of seedlings during the entire study period [55]. However, Millberg et al. [2] found that fungal OTU richness was significantly higher in symptomatic needles (pathogen not specified) compared to healthy needles. In the current work, we did not find any trend in fungal species richness between DNB-symptomatic and asymptomatic trees, irrespective of needle age (*p* > 0.05). 

### 4.3. Relations between Sampling Date, Needle Age, and Location in the Canopy and Relative Abundance of D. septosporum and L. conigenum

DNB infection occurs mainly in the spring and early summer with the aid of rain-dispersed conidia produced by conidiomata formed on infected needles [62]. The first symptoms can appear in autumn, but the characteristic red bands that contain the black conidiomata appear next spring [62]. Infected needles drop during the following summer and early autumn [63]. Similar seasonal processes have been observed in Estonia, where the amount of conidiomata of *D. septosporum* on the attached needles was high in spring, but during the summer almost all the needles with conidiomata were shed [23]. Yet, results of the current study indicated that the relative abundance of *D. septosporum* in pine needles was highest in summer (*p* < 0.05), followed by spring and autumn (except in Konguta site). As *D. septosporum* life cycle includes a substantial epiphytic period during which an extensive web of hyphae is formed on the needle surface and persists for several weeks in the case of *P. radiata* [61], it is reasonable to presume that the pathogen behaves similarly on *P. sylvestris* in northern Europe. During this epiphytic period, no symptomatic red bands appear on needles, which can explain the sequencing-based detection of *D. septosporum* in asymptomatic needles in our study. To determine the length of the epiphytic phase of *D. septosporum* in the case of *P. sylvestris* needles in northern Europe, further studies are needed. Millberg et al. [55] concluded that needle infection by *D. septosporum* may involve an up to12-month-long latent period before symptoms and conidiomata are formed, the length of this time depending on site and local abiotic conditions. 

Generally, needles situated in the lower part of *P. sylvestris* canopies suffer more by *D. septosporum* than needles in the upper part [19]. We also constructed a model to evaluate the relation between the needle canopy position and the relative abundance of *D. septosporum*. Relative abundance of *D. septosporum* was significantly (*p* < 0.05) lower in needles in the upper part of the crown, compared to the lower part. The percentage of *D. septosporum* was significantly higher in second- and third-year needles compared to first-year needles. In 2-month-old needles (2014), the relative abundance of *D. septosporum* was 3.2% and that of *L. conigenum* 4.5%. Needle age was positively related to relative abundance of *D. septosporum* but not to that of *L. conigenum*. 

*Lophodermium conigenum* and *D. septosporum* differed in their seasonal pattern. *Lophodermium conigenum* was extremely abundant in older spring needles in both countries, showing significantly higher relative abundance compared to younger spring needles or older summer or autumn needles (see Supplementary Figure S4). This, in tandem with the higher percentage of *L. conigenum* on senescing needles in comparison to *D. septosporum*, suggests that *L. conigenum*, unlike *D. septosporum*, is a typical endophyte as its colonization level increases along with needle age and senescence. As the fruiting bodies of *L. conigenum* are formed on shed litter in spring, we can presume that feedback from saprobic sporulation contributed to the spring peak in the abundance of this fungus. 

An NGS-based approach is needed to assess the presence and relative abundance of *L. conigenum* on shed needles. Although *L. conigenum* was extremely abundant in second- and third-year needles, it was found in much lower numbers in first-year needles. This shows that the niche is temporally heterogeneous and indicates that *D. septosporum* establishes itself in the niche at a time when there are more resources and space to exploit. As Stachowicz and Tilman [64] theorize relying on data from both grassland plants and marine invertebrates—the more there are empty niches in a community, the easier it is for an invasive species to become established in a new environment. Current-year needles have possibly a lot of empty space, which facilitates establishment by species such as *S. polyspora* and *D. septosporum*, in comparison to second- and third-year needles where species richness and general colonization density of the tissues are higher. Species such as *L. conigenum* may occupy similar niches as *D. septosporum* and reduce the success of establishment by invaders arriving late in the niche. 

Different epiphytes and endophytes have distinct sporulation regimes and possibly prefer tissues with different age indicating temporal complementarity in niche recruitment. Similar to findings from needles of larch [14] and first-year needles of spruce [14,15], some fungal species obviously depend on colonizing first-year needles of *P. sylvestris*, such as species in the rust genus *Coleosporium* [65]. As with *D. septosporum*, *L. conigenum* was more abundant (*p* < 0.05) on lower part of the canopy compared to the upper part where the needles are exposed to harsher environmental conditions, e.g., the frequent, repeated, rapid shifts in humidity, temperature and UV radiation. Such conditions favor fungi that have specific traits to inhabit foliage surfaces. Extremophilic species such as *A. pullulans* (de Bary) G. Arnaud and foliicolous yeasts use production of an extracellular polysaccharide matrix that is thought to facilitate survival and growth in oligotrophic extreme environments. In yeasts, these capsules secure attachment, provide protection against desiccation and allow efficient rehydration following periods of drought, and bind nutrients, thus contributing to higher growth rates of encapsulated yeast cells versus non-capsulated variants on nutrient-poor media [66]. 

### 4.4. The Presence of Other Lophodermium Species in Estonia and Norway

*Lophodermium seditiosum* is known as a causative agent of pine needle-cast epidemics that can last up to 2 years. The low relative abundance of this species in the present study would indicate that the environmental conditions during the study period were not favourable for this pathogen [31]. In Sweden, *L. seditiosum* has been found in both healthy and diseased needles, the former probably representing latent infections [2]. Low numbers of *L. seditiosum* sequences were found in current study in both DNB symptomatic and asymptomatic trees. Of endophytic *Lophodermium* species [29], *L. pinastri* s. str. and *L. conigenum* displayed a higher relative abundance in diseased needles in Sweden compared to our study [2]. In the current study, *L. pinastri* s. str. was relatively abundant as well and, similarly to Sweden, it showed higher relative abundance on DNB symptomatic trees (2.8%) compared to visibly healthy trees (0.6%), even though the difference was not statistically significant (*p* > 0.05). 

### 4.5. Fungal Species Composition 

PERMANOVA analysis showed that species composition was mostly influenced by sampling site, which may relate to site-specific differences in climate, understorey vegetation [38,58] and forest demography as some species can spread also to the foliage of younger trees from nearby older stands [67]. In the current work, differences in fungal species composition were statistically significantly explained by symptoms of DNB on the needles. While a Swedish study [2] showed differences in fungal species composition between healthy and symptomatic needles of Scots pine, they included foliage with several different symptoms (precise agent unknown). On the other hand, Schlegel et al. [58] and Agan et al. [38] indicated no differences in overall fungal species composition between leaves from healthy and diseased trees of ash. In addition, Kovalchuk et al. [68] suggested that there were no significant differences in structure of foliage mycobiome between healthy and symptomatic *Picea abies* trees, but they assessed the overall health condition of the tree and did not consider specific diseases or symptoms separately. 

### 4.6. Co-Occurrence and Pathogen Effects

The relative abundance of *L. conigenum* was negatively associated (*p* < 0.01) with overall fungal species richness in both countries, but this relationship disappeared as overall fungal species richness increased, e.g., in summer or autumn, and it may thus represent a stochastic relation. In different needle-age classes, it became evident that *L. conigenum* had a significant positive relationship with overall fungal species richness on second-yearneedles in Estonia and on second- and third-year needles in Norway. 

The relative abundance of *D. septosporum* showed no significant relation with overall fungal species richness in any of the four sampling sites, or between the three sampling dates or needle-age classes. The fungal community composition did not differ between DNB-symptomatic and DNB-free *P. sylvestris* needles. This provides support to the hypothesis that *D. septosporum* has been co-evolving a long time with *P. sylvestris* and its associated mycobiota [69,70]. 

### 4.7. Technical Notes

As Loit et al. [44] observed, it may be difficult to differentiate between some common pine pathogens, such as *D. septosporum* and *D. pini*, at the most commonly used 97% sequence similarity threshold and even at the 98% sequence similarity threshold. The issue was resolved after raising the similarity threshold up to 99%. In our study, all *D. septosporum* sequences could be reliably identified as *D. pini*. In addition to *Dothistroma* species, we noticed that similar clustering and mislabelling issues concern also *Lophodermium* species, e.g., *L. seditiosum* and *L. pinastri*. At the 97% similarity threshold these species were clustered together as *L. pinastri*. We used a 99% sequence similarity threshold to resolve this issue. We also manually BLAST-ed all identified *D. pini*, *Dothistroma* sp., *L. pinastri*, *L. seditiosum* and *Lophodermium* sp. OTU reference sequences against the UNITE database [45] to reduce the probability of misidentification. These results show how universal approaches that work well in general metabarcoding-based community studies may fall short in identifying and/or differentiating between specific closely related species within these large datasets. Raising the similarity threshold and manually checking specific OTUs of interest is warranted. 

## 5. Conclusions

We conclude that species richness on pine needles increased from spring to autumn and with needle age. Species richness is possibly affected also by temperature sum but examination of a larger number of sites is needed to test this hypothesis. Additionally, fungal species richness was significantly higher in the needles situated in the lower part of tree canopies. It is noteworthy that species richness in second-year needles in Estonia and third-year needles in Norway was statistically similar, suggesting that a critical colonization threshold for pine needle shed is reached in Estonia a year earlier than in Norway, possibly due to warmer climate that stimulates microbial growth. Needle-age class also had a significant positive relationship with species richness. Both *D. septosporum* and *L. conigenum* had higher relative abundance in the lower parts of tree canopies. DNB symptomatic and healthy trees had a similar relative abundance of *D. septosporum*. The relative abundance of *D. septosporum* peaked in summer, while that of *L. conigenum* was highest in spring, reflecting that different dominant species in *P. sylvestris* needles can have different sporulation regimes and niche recruitment times. Relative abundance of *L. conigenum* had a significant negative relation with overall species richness in two- and three-year-old *P. sylvestris* needles in spring. In contrast, relative abundance of *D. septosporum* had no relation with overall species richness of pine needle mycobiome. Fungal species composition was mostly influenced by sampling site, needle age, needle location in canopy and presence of *D. septosporum* symptoms.

## Data Availability

Filtered representative sequences for each sample are available in PlutoF—location: https://dx.doi.org/10.15156/BIO/1619342. All raw sequences are available in SRA (sequence read archive) under accession number: PRJNA735912.

## Supplementary Materials

Supplementary materials are available at https://www.mdpi.com/article/10.3390/microorganisms9081757/s1: Table S1: Abundance of fungal species detected on needles of *Pinus sylvestris* in Estonia (a) and Norway (b). For growth form, F indicates filamentous, Y, yeast and D dimorphic. For the hypothetical functional group, En indicates endophytic, Ep epiphytic, B biotrophic, M mycoparasitic, N necrotrophic and S saprotrophic; Table S2: Species with positive and negative association with *D. septosporum* and *L. conigenum*; Figure S1: The distribution of sampled Scots pine needles across countries, needle age classes and their location on crown on all sampling sites (the number in brackets shows the number of samples); Figure S2: Across season changes in fungal classes present on Scots pine needles across Estonian sampling sites and needle-age classes; Figure S3: Across season changes in fungal classes present on Scots pine needles across Norwegian sampling sites and needle-age classes; Figure S4: Species richness in studied sites in Estonia and Norway in different parts of the canopies and in needles with different age (in months) (a); Relative abundance of *D. septosporum* and *L. conigenum* in needles with different age (in months, Estonian and Norwegian samples combined (b). Figure S5: Overall species richness of *P. sylvestris* needles across four sampling sites, three sampling times and three needle-age cohorts

## References

[1] Terhonen E., Marco T., Sun H., Jalkanen R., Kasanen R., Vuorinen M., Asiegbu F. (2011). The Effect of Latitude, Season and Needle Age on the Mycota of Scots Pine (*Pinus sylvestris*) in Finland. Silva. Fennica.

[2] Millberg H., Boberg J., Stenlid J. (2015). Changes in Fungal Community of Scots Pine (*Pinus sylvestris*) Needles along a Latitudinal Gradient in Sweden. Fungal Ecol..

[3] Taudière A., Bellanger J.-M., Carcaillet C., Hugot L., Kjellberg F., Lecanda A., Lesne A., Moreau P.-A., Scharmann K., Leidel S. (2018). Diversity of Foliar Endophytic Ascomycetes in the Endemic Corsican Pine Forests. Fungal Ecol..

[4] Hyde K., Soytong K. (2008). The Fungal Endophyte Dilemma. Fungal Diversity.

[5] Petrini O., Andrews J.H., Hirano S.S. (1991). Fungal Endophytes of Tree Leaves. Microbial Ecology of Leaves.

[6] Hane J.K., Paxman J., Jones D.A.B., Oliver R.P., de Wit P. (2020). “CATAStrophy,” a Genome-Informed Trophic Classification of Filamentous Plant Pathogens—How Many Different Types of Filamentous Plant Pathogens Are There?. Front. Microbiol..

[7] Clay K., Andrews J.H., Hirano S.S. (1991). Endophytes as Antagonists of Plant Pests. Microbial Ecology of Leaves.

[8] Carrol G. (1995). Forest endophytes: Pattern and process. Can. J. Bot..

[9] Faeth S.H., Wilson D., Gange A.C. (1997). Induced Responses in Trees: Mediators of Interactions among Macro- and Micro-Herbivores?. Multitrophic Interactions in Terrestrial Systems.

[10] Stone J., Petrini O., Carroll G.C., Tudzynski P. (1997). Endophytes of Forest Trees: A Model for Fungus-Plant Interactions. Plant Relationships Part B.

[11] Wilson D., Carroll G.C. (1997). Avoidance of High-Endophyte Space by Gall-Forming Insects. Ecology.

[12] Sieber T.N. (2007). Endophytic Fungi in Forest Trees: Are They Mutualists?. Fungal Biol. Rev..

[13] Delaye L., García-Guzmán G., Heil M. (2013). Endophytes versus Biotrophic and Necrotrophic Pathogens—Are Fungal Lifestyles Evolutionarily Stable Traits?. Fungal Divers..

[14] Gaeumann E. (1959). Die Rostpilze Mitteleuropas.

[15] Johnson J.A., Whitney N.J. (1992). Isolation of Fungal Endophytes from Black Spruce (Picea Mariana) Dormant Buds and Needles from New Brunswick, Canada. Can. J. Bot..

[16] Magan N., Smith M.K., Kirkwood I.A. (1994). Effects of Atmospheric Pollutants on Phyllosphere and Endophytic Fungi. Fungi and environmental change: Symposium of the British Mycological Society, held at Cranfield University.

[17] Deckert R.J., Peterson R.L. (2000). Distribution of Foliar Fungal Endophytes of Pinus Strobus between and within Host Trees. Can. J. For. Res..

[18] Barnes I., Crous P.W., Wingfield B.D., Wingfield M.J. (2004). Multigene Phylogenies Reveal That Red Band Needle Blight of Pinus Is Caused by Two Distinct Species of Dothistroma, D. Septosporum and D. Pini. Stud.Mycol..

[19] Drenkhan R., Tomešová-Haataja V., Fraser S., Bradshaw R.E., Vahalík P., Mullett M.S., Martín-García J., Bulman L.S., Wingfield M.J., Kirisits T. (2016). Global Geographic Distribution and Host Range of Dothistroma Species: A Comprehensive Review. For. Pathol..

[20] Doroguine M. (1911). A Cryptogamic Disease of Pines [In French]. Bull. Trimest. Société Mycol. Fr..

[21] Barnes I., Walla J.A., Bergdahl A., Wingfield M.J. (2014). Four New Host and Three New State Records of Dothistroma Needle Blight Caused by Dothistroma Pini in the United States. Plant Dis..

[22] Bradshaw R.E. (2004). Dothistroma (Red-Band) Needle Blight of Pines and the Dothistromin Toxin: A Review. For. Pathol..

[23] Hanso M., Drenkhan R. (2008). First Observations of Mycosphaerella Pini in Estonia. Plant. Pathol..

[24] Solheim H., Vuorinen M. (2011). First Report of Mycosphaerella Pini Causing Red Band Needle Blight on Scots Pine in Norway. Plant. Dis..

[25] Drenkhan R., Kurkela T., Hanso M. (2006). The Relationship between the Needle Age and the Growth Rate in Scots Pine (*Pinus sylvestris*): A Retrospective Analysis by Needle Trace Method (NTM). Eur. J. For. Res..

[26] Kurkela T., Drenkhan R., Vuorinen M., Hanso M. (2009). Growth Response of Young Scots Pines to Needle Loss Assessed from Productive Foliage. For. Stud. Metsanduslikud Uurim..

[27] Reignoux S.N.A., Green S., Ennos R.A. (2014). Molecular Identification and Relative Abundance of Cryptic Lophodermium Species in Natural Populations of Scots Pine, *Pinus sylvestris*, L.. Fungal Biol..

[28] Stone J.K., Bacon C.W., White J.F. (2000). An Overview of Endophytic Microbes: Endophytism Defined. Microbial Endophytes.

[29] Minter D.W., Millar C.S. (1980). Ecology and biology of three Lophodermium species on secondary needles of *Pinus sylvestris*. Eur. J. For. Res..

[30] Diwani S.A., Millar C.S. (1987). Pathogenicity of Three Lophodermium Species on *Pinus sylvestris*, L.. Eur. J. For. Res..

[31] Hanso M., Drenkhan R. (2012). Lophodermium Needle Cast, Insect Defoliation and Growth Responses of Young Scots Pines in Estonia. For. Pathol..

[32] Dietrich H.A. (1856). Blicke in die cryptogamenwelt der Ostseeprovinzen.

[33] Jansons Ā., Zeltiņš P., Donis J., Neimane U. (2020). Long-Term Effect of Lophodermium Needle Cast on The Growth of Scots Pine and Implications for Financial Outcomes. Forests.

[34] Woods A., Coates K.D., Hamann A. (2005). Is an Unprecedented Dothistroma Needle Blight Epidemic Related to Climate Change?. BioScience.

[35] Lõhmus E. (2004). Eesti Metsakasvukohatüübid [In Estonian].

[36] Tedersoo L., Anslan S. (2019). Towards PacBio-based pan-eukaryote metabarcoding using full-length ITS sequences. Environ. Microbiol. Rep..

[37] Tedersoo L., Bahram M., Põlme S., Kõljalg U., Yorou N.S., Wijesundera R., Ruiz L.V., Vasco-Palacios A.M., Thu P.Q., Suija A. (2014). Global Diversity and Geography of Soil Fungi. Science.

[38] Agan A., Drenkhan R., Adamson K., Tedersoo L., Solheim H., Børja I., Matsiakh I., Timmermann V., Nagy N.E., Hietala A.M. (2020). The Relationship between Fungal Diversity and Invasibility of a Foliar Niche—The Case of Ash Dieback. J. Fungi.

[39] Schloss P.D., Westcott S.L., Ryabin T., Hall J.R., Hartmann M., Hollister E.B., Lesniewski R.A., Oakley B.B., Parks D.H., Robinson C.J. (2009). Introducing Mothur: Open-Source, Platform-Independent, Community-Supported Software for Describing and Comparing Microbial Communities. Appl. Environ. Microbiol..

[40] Edgar R.C., Haas B.J., Clemente J.C., Quince C., Knight R. (2011). UCHIME Improves Sensitivity and Speed of Chimera Detection. Bioinformatics.

[41] Bengtsson-Palme J., Ryberg M., Hartmann M., Branco S., Wang Z., Godhe A., De Wit P., Sánchez-García M., Ebersberger I., de Sousa F. (2013). Improved software detection and extraction of ITS1 and ITS2 from ribosomal ITS sequences of fungi and other eukaryotes for analysis of environmental sequencing data. Methods Ecol. Evol..

[42] Fu L., Niu B., Zhu Z., Wu S., Li W. (2012). CD-HIT: Accelerated for Clustering the next-Generation Sequencing Data. Bioinformatics.

[43] Anslan S., Bahram M., Hiiesalu I., Tedersoo L. (2017). PipeCraft: Flexible Open-Source Toolkit for Bioinformatics Analysis of Custom High-Throughput Amplicon Sequencing Data. Mol Ecol Resour..

[44] Loit K., Adamson K., Bahram M., Puusepp R., Anslan S., Kiiker R., Drenkhan R., Tedersoo L. (2019). Relative Performance of Oxford Nanopore MinION vs. Pacific Biosciences Sequel Third-Generation Sequencing Platforms in Identification of Agricultural and Forest Pathogens. Appl. Environ. Microbiol..

[45] Kõljalg U., Nilsson R.H., Abarenkov K., Tedersoo L., Taylor A.F.S., Bahram M., Bates S.T., Bruns T.D., Bengtsson-Palme J., Callaghan T.M. (2013). Larsson, K.-H. Towards a Unified Paradigm for Sequence-Based Identification of Fungi. Mol. Ecol..

[46] Tedersoo L., Drenkhan R., Anslan S., Morales-Rodriguez C., Cleary M. (2019). High-throughput identification and diagnostics of pathogens and pests: Overview and practical recommendations. Mol. Ecol. Resour..

[47] Hammer Ø., Harper D.A.T., Ryan P.D. (2001). PAST: Paleontological Statistics Software Package for Education and Data Analysis. Palaeontologia Electronica.

[48] Bates D., Mächler M., Bolker B., Walker S. (2014). Fitting Linear Mixed-Effects Models Using Lme4. J. Stat. Softw..

[49] Clarke K.R., Gorley R.N. (2006). PRIMER v6: User Manual/Tutorial.

[50] Anderson M., Gorley R.N., Clarke R.K. (2008). Permanova+ for Primer: Guide to Software and Statistical Methods.

[51] Bray J.R., Curtis J.T. (1957). An Ordination of the Upland Forest Communities of Southern Wisconsin. Ecol. Monogr..

[52] Lazarević J., Menkis A. (2020). Fungal Diversity in the Phyllosphere of Pinus Heldreichii, H. Christ—An Endemic and High-Altitude Pine of the Mediterranean Region. Diversity.

[53] Minter D.W. (1981). Possible Biological Control of Lophodermium Seditiosum. Current Research on Conifer Needle Diseases, Proceedings of IUFRO WP on Needle Diseases, Sarajevo, 1980.

[54] Müller M.M., Hantula J., Vuorinen M. (2009). First Observations of Mycosphaerella Pini on Scots Pine in Finland. Plant. Dis..

[55] Millberg H., Hopkins A.J.M., Boberg J., Davydenko K., Stenlid J. (2016). Disease development of Dothistroma needle blight in seedlings of *Pinus sylvestris* and Pinus contorta under Nordic conditions. For. Pathol..

[56] Liu C.M., Kachur S., Dwan M.G., Abraham A.G., Aziz M., Hsueh P.-R., Huang Y.-T., Busch J.D., Lamit L.J., Gehring C.A. (2012). FungiQuant: A Broad-Coverage Fungal Quantitative Real-Time PCR Assay. BMC Microbiol..

[57] Jumpponen A., Jones K.L. (2010). Seasonally Dynamic Fungal Communities in the Quercus Macrocarpa Phyllosphere Differ between Urban and Nonurban Environments. New Phytol..

[58] Schlegel M., Queloz V., Sieber T.N. (2018). The Endophytic Mycobiome of European Ash and Sycamore Maple Leaves—Geographic Patterns, Host Specificity and Influence of Ash Dieback. Front. Microbiol..

[59] Peršoh D. (2015). Plant-Associated Fungal Communities in the Light of Meta’omics. Fungal Divers..

[60] Zamora P., Martínez-Ruiz C., Diez J.J. (2008). Fungi in Needles and Twigs of Pine Plantations from Northern Spain. Fungal Divers..

[61] Kabir M.S., Ganley R.J., Bradshaw R.E. (2015). The Hemibiotrophic Lifestyle of the Fungal Pine Pathogen Dothistroma Septosporum. For. Pathol..

[62] Gibson I.A.S. (1972). Dothistroma Blight of Pinus Radiata. Annu. Rev. Phytopathol..

[63] Karadžič D. (1989). Scirrhia Pini Funk et Parker. Life Cycle of the Fungus in Plantations of Pinus Nigra Arn. in Serbia. Eur. J. Plant Pathol..

[64] Stachowicz J., Tilman D. (2005). Species Invasions and the Relationships between Species Diversity, Community Saturation, and Ecosystem Functioning 2. Species Invasions: Insights into Ecology, Evolution, and Biogeography.

[65] Lynikienė J., Marčiulynienė D., Marčiulynas A., Gedminas A., Vaičiukynė M., Menkis A. (2020). Managed and Unmanaged *Pinus sylvestris* Forest Stands Harbour Similar Diversity and Composition of the Phyllosphere and Soil Fungi. Microorganisms.

[66] Golubev W.I., Rose A.H., Harrison J.S. (1991). Capsules. The Yeasts.

[67] Arnold A.E., Herre E.A. (2003). Canopy Cover and Leaf Age Affect Colonization by Tropical Fungal Endophytes: Ecological Pattern and Process in Theobroma Cacao (Malvaceae). Mycologia.

[68] Kovalchuk A., Mukrimin M., Zeng Z., Raffaello T., Liu M., Kasanen R., Sun H., Asiegbu F.O. (2018). Mycobiome Analysis of Asymptomatic and Symptomatic Norway Spruce Trees Naturally Infected by the Conifer Pathogens Heterobasidion spp.. Environ. Microbiol. Rep..

[69] Adamson K., Mullett M.S., Solheim H., Barnes I., Müller M.M., Hantula J., Vuorinen M., Kačergius A., Markovskaja S., Musolin D.L. (2018). Looking for Relationships between the Populations of Dothistroma Septosporum in Northern Europe and Asia. Fungal Genet. Biol..

[70] Mullett M.S., Drenkhan R., Adamson K., Boroń P., Lenart-Boroń A., Barnes I., Tomšovský M., Jánošíková Z., Adamčíková K., Ondrušková E. (2021). Worldwide Genetic Structure Elucidates the Eurasian Origin and Invasion Pathways of Dothistroma Septosporum, Causal Agent of Dothistroma Needle Blight. J. Fungi.

