# Supplementary material for: Seasonal Dynamics of Fungi Associated with Healthy and Diseased Pinus sylvestris Needles in Northern Europe
Source: Microorganisms. 2021 Aug 17;9(8):1757. doi: 10.3390/microorganisms9081757 (PMC8400686; doi:10.3390/microorganisms9081757)
Supplement: Supplementary file 1 [file microorganisms-09-01757-s001.zip › microorganisms-1345783-supplementary.pdf]

Supplementary materials

Table S1. Abundance of fungal species detected on needles of *Pinus sylvestris* in Estonia (a) and Norway (b). For growth form, F indicates filamentous, Y, yeast and D dimorphic. For the hypothetical functional group, En indicates endophytic, Ep epiphytic, B biotrophic, M mycoparasitic, N necrotrophic and S saprotrophic

(a)

Growth form	Hypothetical Functional group	Species	UNITE species hypotheses	Percentage
F	En	*Lophodermium conigenum*	SH2376405.08FU	19.36
	En	*Sydowia polyspora*	SH021773.07FU	7.64
		Capnodiales sp	SH017690.07FU	5.56
	B	*Coleosporium sp*		4.67
F	En	*Lophodermium pinastri*	SH000656.07FU	4.30
Y	-	*Phaeococcomyces sp*		4.24
	Ep/En	*Dothistroma septosporum*	SH022044.07FU	3.74
-	-	*Dothidemycetes sp1*		3.41
		Cladosporium sp		2.78
F	B	*Exsobasidium sp*		2.48
-	-	*Fungi sp1.*		2.25
-	-	*Lapidomyces sp*		2.12
F	B	*Exobasidium sp*		1.69
		Ascomycota sp		1.59
Y		*Malassezia globosa*	SH011745.07FU	1.36
D		*Tremellomycetes sp.*		1.29
D	Ep/En	*Aureobasidium pullulans*	SH014188.07FU	1.09
F	B	*Exobasidium vaccinii*	SH025676.07FU	0.99
		Dothideomycetes sp2		0.96
-	-	*Fungi sp2.*		0.93

(b)

Growth form	Hypothetical functional group	Species	UNITE species hypotheses	Percentage
F	En	*Lophodermium conigenum*	SH2376405.08FU	21.84
	En	*Phacidiaceae* sp	SH013364.07FU	16.19
	Ep/En	*Dothistroma septosporum*	SH022044.07FU	6.60
		Capnodiales sp	SH017690.07FU	5.20
	En	*Sydowia polyspora*	SH021773.07FU	4.31
	S	*Phacidium lacerum*	SH013364.07FU	3.11
		Mycosphaerellaceae sp		2.97
-	-	Fungi sp1		2.13
		Scleroconidioma sp		1.93
D		*Tremellomycetes* sp.		1.91
-	-	Fungi sp2		1.79
-	-	Fungi sp3		1.79
	S	*Claussenomyces* sp		1.63
		Ascomycota sp		1.58
-	-	*Agaricomycetes* sp		1.50
-	-	*Helotiales* sp		1.44
Y	-	*Curvibasidium* sp		1.26
F	En	*Lophoderimium* sp		1.24
		Phacidiaceae sp		1.04
		Fungi sp. 4		1.02

Table S2. Species with positive and negative association with *D. septosporum* and *L. conigenum.*

D. Septosporum				*L. conigenum*	
**Positive association**	***p*-value**	**Negative association**	**P-value**	**Positive association**	***p*-value**
Ascomycota sp.	6.00×10^{-5}	*Chaetothyriales* sp.	0.00753	*Lophodermium* sp.	3.00×10^{-5}
Mycosphaerellaceae sp.	8.00×10^{-5}	*Malassezia restricta*	0.02651	*Tremellomycetes* sp.	0.00028
Curvibasidium sp.	0.00017			*Agaricomycetes* sp.	0.00038
Fungi sp.	0.00072			*Capnodiales* sp.	0.00095
Scleroconidioma sp.	0.00149			Fungi sp.	0.00138
Exsobasidium sp.	0.00175			*Dothistroma septosporum* 1	0.00217
Tremellomycetes sp.	0.00297			Fungi sp.	0.00236
Fungi sp.	0.0038			*Lophodermium* sp.	0.0034
Agaricomycetes sp.	0.0043			*Phacidiaceae* sp	0.0034
Lophoderimium sp.	0.00469			Fungi sp.	0.00543
Dothistroma septosporum 2	0.00469			*Scleroconidioma* sp	0.00637
Fungi sp.	0.0087			Fungi sp.	0.01083
Sarocladium strictum	0.014			*Phacidiaceae* sp.	0.01083
Fungi sp.	0.014			Fungi sp.	0.01083
Lophoderimium sp.	0.014			*Lophoderimium* sp.	0.01083
Helotiales sp.	0.02037			*Helotiales* sp	0.01512
Cryptococcus victoriae	0.02037			*Phacidium lacerum*	0.01605
Symmetrospora gracilis	0.02037			Genolevuria sp	0.01605
Capnobotryella sp.	0.02254			Fungi sp.	0.02438
Rhodosporidiobolus colostri	0.02254			*Tremella* sp.	0.02683
Genolevuria sp.	0.02254			*Venturia* sp.	0.02683
Dothidemycetes sp.	0.02457			*Dothidemycetes* sp.	0.02748
Fellomyces sp.	0.03154			*Mycosphaerellaceae* sp.	0.03394
Dothideomycetes sp.	0.0399			Fungi sp.	0.0356
Tremella sp.	0.0399			*Lapidomyces* sp.	0.04024
Phaeomoniella sp.	0.04132			*Exobasidium* sp.	0.04024
Fungi sp.	0.04132			*Phacidiaceae* sp.	0.04058
Capnobotryella sp.	0.04132			*Ceramothyrium* sp.	0.04058
Lophoderimium sp.	0.04877			*Dothistroma septosporum* 2	0.04058
				Dothideomycetes sp.	0.04605
				Fungi sp.	0.04906

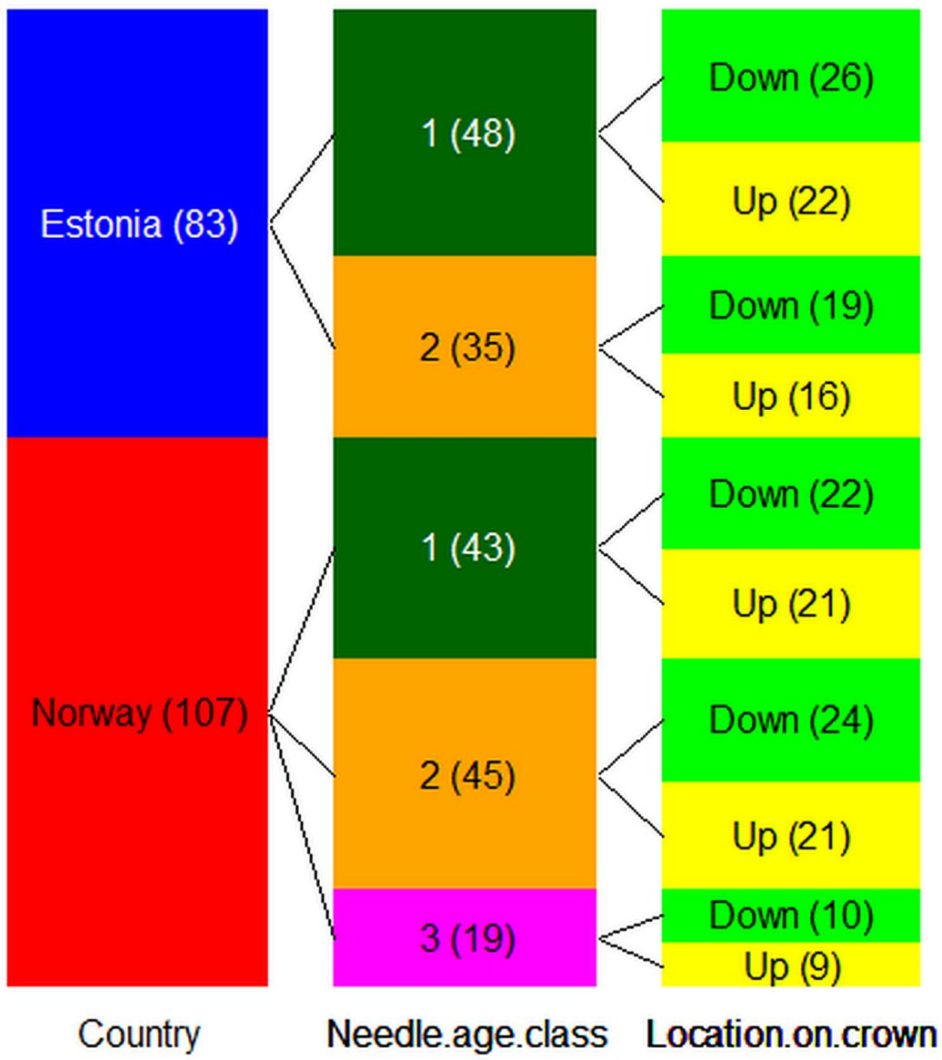

Figure S1. The distribution of sampled Scots pine needles across countries, needle age classes and their location on crown on all sampling sites (number in brackets shows the number of samples).

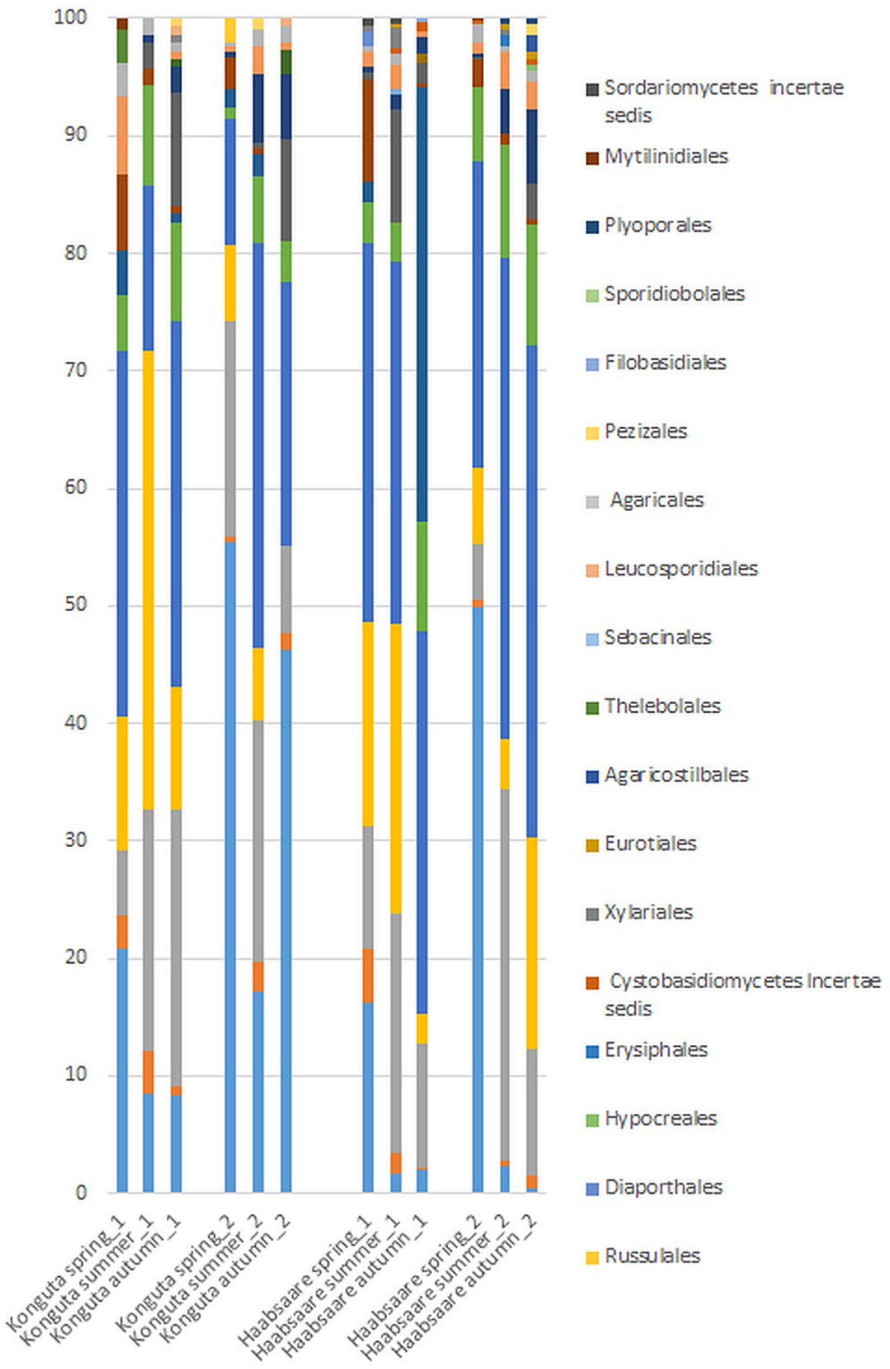

Figure S2. Across season changes in fungal classes present on Scots pine needles across Estonian sampling sites and needle-age classes

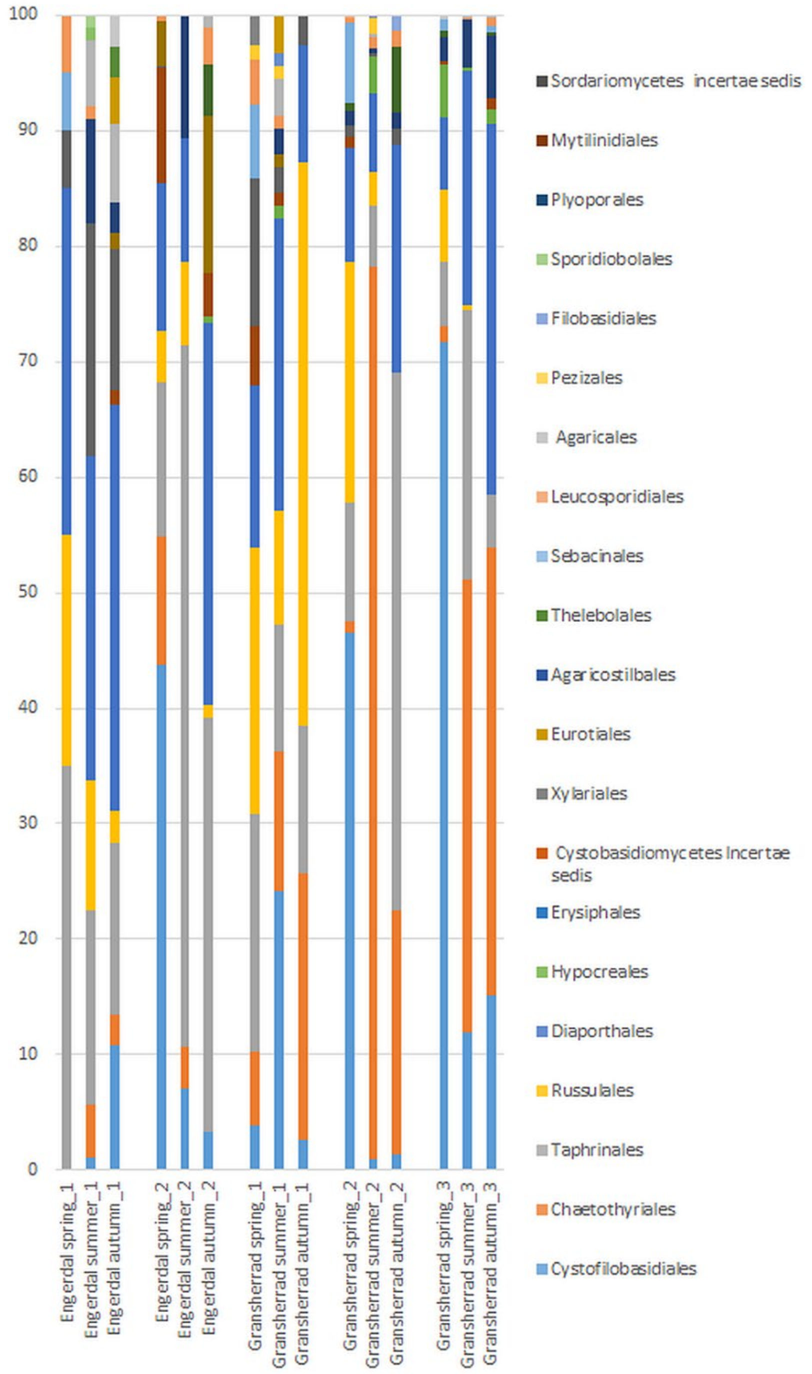

Figure S3. Across season changes in fungal classes present on Scots pine needles across Norwegian sampling sites and needle-age classes

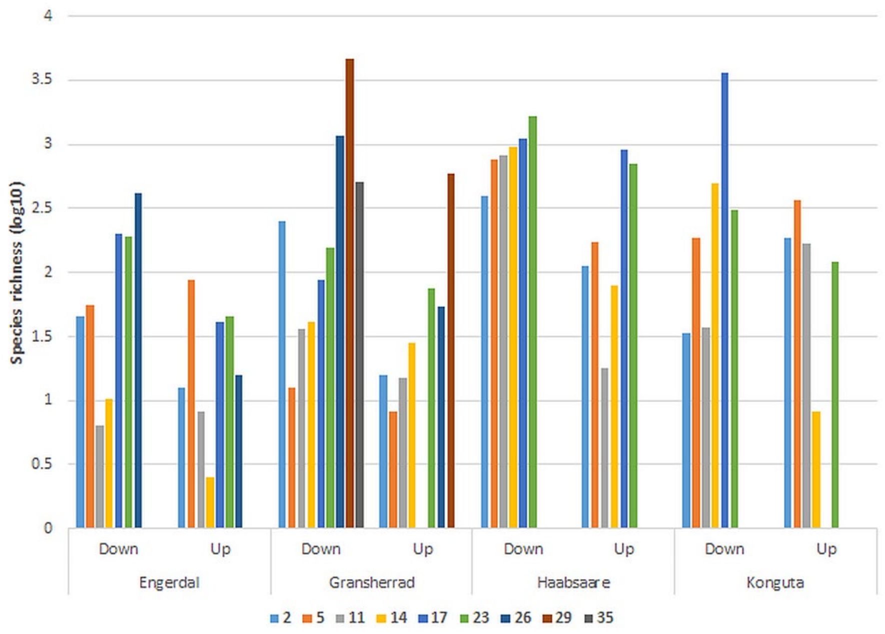

(a)

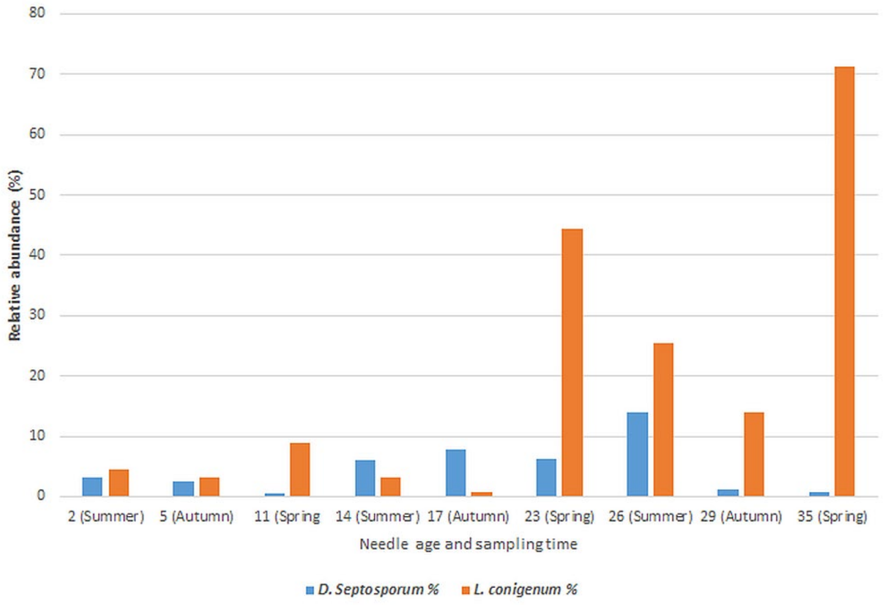

(b)

Figure S4. Species richness in studied sites in Estonia and Norway in different parts of the canopies and needles with different needle age (in months) (**a**); Relative abundance of *D. septosporum* and *L. conigenum* in needles with different age (in months, Estonian and Norwegian samples combined (**b**).

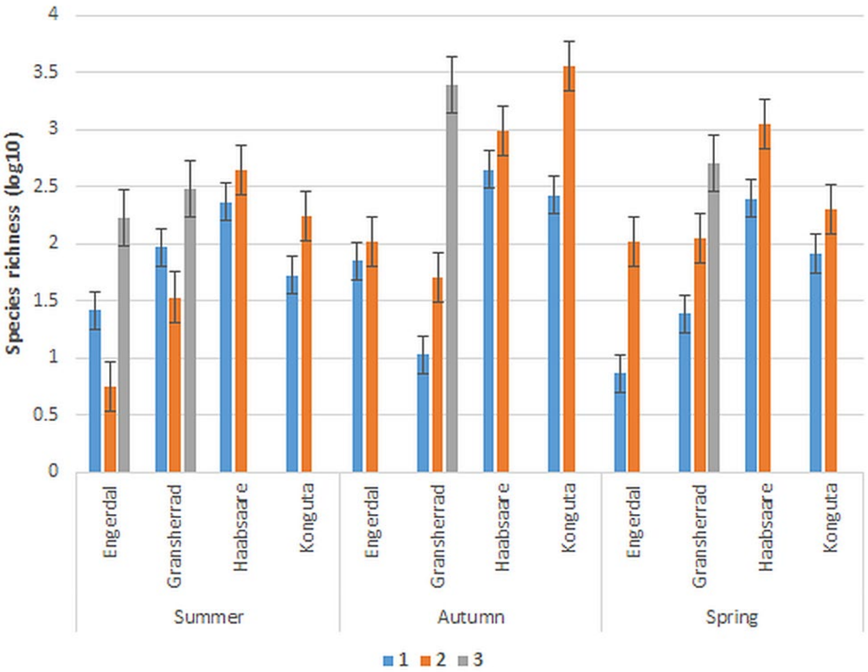

Figure S5. Overall species richness of *P. sylvestris* needles (N=190) across four sampling sites, three sampling times and three needle-age cohorts. Whiskers show standard error.
